# The Dentist as a Prescriber of Drugs

**Published:** 1893-08

**Authors:** Edward C. Briggs

**Affiliations:** Boston


### THE DENTIST AS A PRESCRIBER OF DRUGS.[1]

BY EDWARD C. BRIGGS, M.D., D.M.D., BOSTON.

In casting about for some subject to present to you to-night, to which creditably or otherwise I might add some original thought, it occurred to me that in Dr. Taft's article, published in the International Dental Journal for September, 1892, there was much food for discussion and suggestion. In this article he advocated, first, enlarging the field of the dentist by including with his other treatment the systemic dosing of the patient; second, the use of the homœopathic high-potency method ; third, that the practice of medicine according to that method is an exact science.

In regard to the first proposition, it seems to the writer that Dr. Taft is leading us into very dangerous ground, and one that, with the average education of the dentist of to-day, is entirely untenable.

While we all like to feel that dentistry in its broadest and noblest sense is a part of the great art of medicine, and not merely expert jewellers' work, it is allied more closely with the other surgical specialties, as the eye, the ear, or general surgery, and like them displays its medical affinity rather by intelligent co-operation with the family physician than by usurpation of his privileges. Even if the dentist were a full graduate in medicine, as most of us are not, he is scarcely competent to take charge of the constitutional treatment of the patient, if for no other reason than because the constant and full demands upon his time for active surgical treatment at his office prevent him from following up his cases as closely and accurately as necessary if he is to carry out a line of logical systemic treatment. How is it, then, with the dental practitioner who is not a graduate of medicine? Is he by buying and perusing one text-book on the treatment of disease to become competent to practice general medicine? Of what use, then, are medical schools, and why should not every intelligent layman buy a book and dismiss the doctor ?

In the Harvard Dental School, where the subject of drugs and their application to disease is gone into to perhaps a fuller extent than in any other dental school, the students are taught that all the collateral instruction is given them, not that they should take up general practice, but that they should have an intelligent

---

[1] Read before Harvard Odontological Society, March 30, 1893.

knowledge of what auxiliaries they may have in their treatment and when and under what circumstances they can call upon the general practitioner to aid them in producing a cure. To be sure, they are taught, and expected to use, the application of many drugs in the class of cases which might be called emergency cases, as, for instance, the use of cardiac stimulants in case of syncope, whether from nervous fright or the use of anæsthetics; but when it comes to the prescribing of drugs to be taken by the patient at his house, away from the care of the dentist, it seems to me we should stop to consider carefully what it means.

The dentist at present occupies an enviable position, in that he is free from the trammels put upon the followers of the different paths or schools in medicine. He treats all alike, whether his patient is a Christian scientist, a believer in mind cure, a high-dilution homœopath, a broad homœopath, an eclectic, an allopath, or a "regular." He has the satisfaction of doing positive helpful work for his patients and seeing that it is good, but Heaven help him if he has to reach out after his patients in their homes, and build this one up with prayer, the next with mental balm, the next with the one-thousandth potency, and perhaps have another who is only satisfied when he has taken the largest dose possible, and escape toxic symptoms.

Do we want to encourage patients to come to us because we are practising in one of these various schools? Taking our patients as they come, have we any more right to dawdle them along with the one-thousandth potency of silica than to salivate them with mercury, especially when we can cure them by the positivism of surgery?

Nor does the course which the writer of this paper advocates preclude a wide and vast knowledge of drugs and other remedies and their relation to disease. On the contrary, he should be able to recognize when his patient needs help outside of his, the dentist's, local treatment, and, by intelligently calling the physician's attention to the need of the case, procure for the patient what is required. Let us be thankful that the patient decides by his choice of a physician whether hypnotism or a course of iron will best suit his case. Suffice for us to know that statistics show that as many get well under one ism as another.

We have, as it is, a grand field for work and helpfulness to our patients, conflicting with no school or ism,—our surgical operations, our topical remedies (which even the Christian scientist does not cavil at), the laws of hygiene and diet, to which all schools of medicine have to bow. Given the fullest knowledge of hygiene and diet, which has not yet been attained by any one, all physic can be thrown to the dogs, even the one-thousandth potency, and we can have that grandest of all spectacles, a physician who cures without drugs.

In regard to Dr. Taft's proposition that medicine is an exact science, the writer ventures to contradict it flatly.

Medicine as practised by any school is not an exact science.

The Hahnemannian law, which is a law founded upon the supposed relation of a drug to a disease, and therefore considered by many an irrational, illogical law, since as such there can be no relation, is really founded on the rule of drugs themselves, which is, that small medicinal doses have a contrary physiological effect to full toxic doses, and therefore a small dose will often antagonize pathological conditions which resemble the symptoms produced by the toxic dose; but this rule, like all others, has its exceptions, and woe betide the physician who lashes himself irrevocably to the spar of that belief.

All these different creeds are steps, and only steps, towards a perfection of healing which is still a long way off.

Lest in my antagonism to some of the principles of Dr. Taft's paper I should seem to be decrying homœopathy, let me pay it this tribute,—it has helped medical practitioners as a whole towards that rational and wise doctrine that obtains to-day, that generally a smaller dose of medicine, often repeated, is better than a larger one at greater intervals, and that we should seek to get our effects with the smallest dose possible. Even this, however, would have come, and did come in many instances, by the rational deductions from the experiences of able men not students of homœopathy, and homœopathy must not claim too much for itself, or it will be in the ridiculous position of a political party which claims all the prosperity of a growing, fruitful country, full of natural resources, to be due to its own wise administrations.

The art of healing with drugs is bound to develop, whether hindered or helped by different isms; but ancient as it is, so long a-growing, it is nevertheless true to-day that surgery stands head and shoulders above it, and we as dental surgeons must remember that when we pause in our surgery to give drugs we are rather looking backward and taking much needful time better devoted to positive surgical operation.

Now, this cutting off of the dentist from the systemic treatment of his patient with drugs still leaves him a field in the study of remedies larger than any one of us will ever be able to cover to his satisfaction. Take the topical remedies which he is called upon to use, what a list they comprise! and what a knowledge of chemistry he ought to have to know when and why he should use them! The principles which underlie his use of them, the possibilities and probabilities of absorption into the system, their antagonists, synergists, and compatibilities.

And, lastly, hygiene and diet. Here, indeed, is meat for strong men. Here is a legitimate field for us, and herein are possibilities which as yet we have not even guessed at. In food preparations we have remedies which are not drugs, and remedies which, if rightly applied, will do more to make our work perfect and keep it so than the use of drugs by any system can do.

In studying food values we can learn how best to supply the phosphates to be used in formation of the teeth, and thus in early life construct teeth that will resist decay; we can regulate the diet to prevent the fermentative processes which make the mucous membrane a hot-bed for germs. We may find out how to so feed our patients as to prevent the inflammatory conditions which result in pyorrhœa alveolaris and kindred complaints; we may supply through the food the various elements of the gastric juice, which may be deficient; we can see to it that oxygen is supplied for the proper oxidation of food ingested, and that children in puberty are not physically destroyed in the attempt to force their brains.

Dentistry is a specialty of medicine, not of a particular medical school, and the dentist of the future will have need for all that the best medical schools can teach him, and a great deal more, and yet be able to practise free from the trammels of any particular doctrine.

